# Suberoylanilide Hydroxamic Acid (SAHA) Treatment Reveals Crosstalk Among Proteome, Phosphoproteome, and Acetylome in Nasopharyngeal Carcinoma Cells

**DOI:** 10.3389/fgene.2022.873840

**Published:** 2022-05-03

**Authors:** Huichao Huang, Ying Fu, Yankun Duan, Ye Zhang, Miaolong Lu, Zhuchu Chen, Maoyu Li, Yongheng Chen

**Affiliations:** ^1^ Department of Infectious Disease, XiangYa Hospital, Central South University, Changsha, China; ^2^ Department of Oncology, NHC Key Laboratory of Cancer Proteomics, XiangYa Hospital, Central South University, Changsha, China; ^3^ Department of Gastroenterology, XiangYa Hospital, Central South University, Changsha, China; ^4^ National Clinical Research Center for Geriatric Disorders, XiangYa Hospital, Central South University, Changsha, China

**Keywords:** suberoylanilide hydroxamic acid (SAHA), proteome, acetylome, phosphoproteome, multi-omics, nasopharyngeal carcinoma (NPC)

## Abstract

Suberoylanilide hydroxamic acid (SAHA), a famous histone deacetylase (HDAC) inhibitor, has been utilized in clinical treatment for cutaneous T-cell lymphoma. Previously, the mechanisms underlying SAHA anti-tumor activity mainly focused on acetylome. However, the characteristics of SAHA in terms of other protein posttranslational modifications (PTMs) and the crosstalk between various modifications are poorly understood. Our previous work revealed that SAHA had anti-tumor activity in nasopharyngeal carcinoma (NPC) cells as well. Here, we reported the profiles of global proteome, acetylome, and phosphoproteome of 5–8 F cells upon SAHA induction and the crosstalk between these data sets. Overall, we detected and quantified 6,491 proteins, 2,456 phosphorylated proteins, and 228 acetylated proteins in response to SAHA treatment in 5–8 F cells. In addition, we identified 46 proteins exhibiting both acetylation and phosphorylation, such as WSTF and LMNA. With the aid of intensive bioinformatics analyses, multiple cellular processes and signaling pathways involved in tumorigenesis were clustered, including glycolysis, EGFR signaling, and Myc signaling pathways. Taken together, this study highlighted the interconnectivity of acetylation and phosphorylation signaling networks and suggested that SAHA-mediated HDAC inhibition may alter both acetylation and phosphorylation of viral proteins. Subsequently, cellular signaling pathways were reprogrammed and contributed to anti-tumor effects of SAHA in NPC cells.

## Introduction

Histone deacetylases (HDACs) modulate gene expressions epigenetically *via* affecting protein acetylation status under diverse situations, thus playing key roles in pathophysiological processes (Shahbazian and Grunstein, 2007). Since HDACs as well as protein acetylation contribute to tumorigenesis, specific inhibitors (HDACi)-targeted HDACs have become a promising approach to treat tumors (Wagner et al., 2010). Suberoylanilide hydroxamic acid (SAHA, vorinostat), a member of the HDACi family, has been used clinically as first-line therapy for refractory cutaneous T-cell lymphomas (CTCL) (Duvic et al., 2007; Marks and Breslow, 2007). In addition to CTCL, more and more research studies have unveiled that SAHA also deploys anti-tumor activities in many other tumors, such as lung cancer, breast cancer, and ovarian cancer as well as head and neck tumors (Munster et al., 2001; Komatsu et al., 2006; Konstantinopoulos et al., 2014; Xu et al., 2014; Wu et al., 2015). However, SAHA’s anti-tumor property toward NPC cells is still elusive.

Protein posttranslational modifications (PTMs) are universal means to maintain fundamental biological functions in the body, including but not limited to gene expression, signal transduction, and cell proliferation (Blixt et al., 2004; Krueger and Srivastava, 2006; Hoffman et al., 2008). Phosphorylation, acetylation, methylation, glycosylation, and ubiquitination are common PTMs. The contribution of PTMs to tumorigenesis and progression has been well reported (Sheehan et al., 2005; Krueger and Srivastava, 2006). Phosphorylation refers to the attachment of the phosphoryl group on the target amino acid and is highly regulated, which is considered the most abundant PTM in eukaryotes. The opposing process is dephosphorylation, and the balance between them is critical for many cellular processes in biology. For example, protein phosphorylation activates or deactivates almost half of the enzymes present in *Saccharomyces cerevisiae*, thereby regulating their functions (Oliveira and Sauer, 2012; Tripodi et al., 2015; Vlastaridis et al., 2017). On the other hand, acetylation describes the process that transfers acetyl moiety from acetyl-CoA to its amino groups in lysine residues, which is enzymatically reversible and is tightly regulated by metabolism-dependent mechanisms. Acetylation and deacetylation interplay is the key to lots of important cellular processes. Thus, malfunctioning of this machinery can result in severe conditions such as cancer, neurodegenerative diseases, and cardiovascular disorders (Drazic et al., 2016). It also has been revealed that acetylation and phosphorylation are closely linked and affect each other. Habibian and Ferguson (2018) reported that HDACi regulated crosstalk between acetylation and phosphorylation in the treatment of cardiac disease. Previous works showed that tau protein underwent acetylation, phosphorylation, and ubiquitination in the development of neurodegenerative diseases (Park et al., 2018). Some studies revealed that both acetylation and phosphorylation occurred in p53 and regulated its activity and stability. For example, phosphorylation of p53 at Ser15 and Ser46 leads to the acetylation of p53 at Lys 382, which in turn induced cell apoptosis and cell cycle arrest (Kishi et al., 2001).

In view of the extensive mediated roles of PTMs and the close relationship between acetylation and phosphorylation, it is reasonable to explore the potential property of SAHA’s anti-tumor activity in NPC cells via PTMs and the crosstalk among diverse protein modifications. Moreover, knowing the characteristics of SAHA in NPC cells is also consistent with the paradigm of precise medicine, which is the integral strategy in healthcare (Golubnitschaja et al., 2013; Seredin et al., 2018). In the current work, we combined TMT labeling, antibody affinity enrichment, and high-resolution LC-MS/MS approaches to quantitatively compare the global proteome, phosphoproteome, and acetylome in 5–8 F cell line with/without SAHA treatment. In addition, bioinformatic-based systematic analyses were applied to investigate the crosstalk among these three protein modifications. Collectively, we demonstrated that SAHA treatment dramatically regulated global proteome, phosphoproteome, and acetylome in the 5–8 F cell line. Moreover, our results showed that some key signaling pathways and cellular metabolic processes as well as widespread protein–protein interactions were modulated upon SAHA treatment via altering phosphorylation and acetylation. Overall, this study provided a novel insight into how SAHA exerted biological functions in NPC cells and presented the scientific data for effectively predictive and personalized treatment of NPC patients.

## Materials and Methods

### Cell Culture

Cells were all purchased from the American Type Culture Collection and cultured as previously described. Briefly, 5–8 F and HNE3 cells were cultured in an RPMI-1640 medium containing 10% fetal bovine serum (FBS) and 1% penicillin and streptomycin in a humidified environment at 37°C and 5% CO_2_.

### Co-Immunoprecipitation (Co-IP) and Immunoblotting

NPC cells were harvested and whole-cell lysates (WCLs) were prepared with NP40 buffer (50 mM Tris–HCl, pH 7.4; 150 mM NaCl; 1% NP-40; and 5 mM EDTA) supplemented with 20 mM β-glycerophosphate and 1 mM sodium orthovanadate. WCLs were then sonicated, centrifuged, and pre-cleared with Sepharose 4B for 1 h. Pre-cleared samples were incubated with indicated antibodies overnight and protein A/G agarose for 1 h at 4°C. Agarose beads were washed extensively, and samples were eluted with SDS-PAGE loading buffer at 95°C for 10 min. The precipitated proteins were analyzed by SDS-PAGE and immunoblotting.

The following primary antibodies were commercially obtained: pan anti-acetyl-lysine (Kac) antibodies (PTM Biolabs, 1:3,000 working dilution), LMNA (Proteintech, 10298-1-AP; 1:1,000 working dilution), p-LMNA ser390 (Affinity, AF3753; 1:1,000 working dilution), WSTF (Cell Signaling Technology, 2,152; 1:1,000 working dilution), phosphoserine monoclonal antibody (ImmunoWay, 5B12; 1:1,000 working dilution), protein A/G agarose (Santa Cruz Biotechnology, sc-2003), and ACTB (Sigma-Aldrich, A5441; 1:10,000 working dilution). ImageJ software (version 1.45s) was used to quantify the gray value of the Western blot results. The Western blot image was digitized to calculate mean ± SD with Student’s t-test (*p* < 0.05).

### Protein Extraction and Digestion

Cells were harvested, then washed with ice-cold PBS, and lysed by incubation in SDS lysis buffer. After quantification, protein digestion was performed according to the filter-aided sample preparation (FASP) procedure. Briefly, 200 ug proteins were reduced with 100 mM DTT, and then 200 ul UA buffer (8 M Urea and 150 mM Tris–HCl, pH 8.0) was added. The mixture was then loaded into a Microcon Ultracel YM-10 filtration device and centrifuged at 14,000 × g for 15 min. The concentrates were then diluted with 200ul UA buffer and centrifuged at 14,000 × g for 15 min. After centrifugation, the concentrates were alkylated in 100ul IAA (50 mM IAA in UA) for 30 min in dark. After centrifuged for 10 min, the concentrates were washed twice with UA buffer and twice with 100 mM NH_4_HCO_3_. Subsequently, trypsin solution (8 μg trypsin in 40 μl NH_4_HCO_3_ buffer) was added to the filter, and proteins were incubated at 37°C overnight. Tryptic peptides were collected by centrifugation followed by an additional wash with elution solution (70% ACN and 0.1% formic acid). Finally, the peptide mixture was desalted with a C18-SD Extraction Disk Cartridge, and the peptide concentration was assayed by measuring absorbance at 280 nm.

### Tandem Mass Tagging Labeling

Three control samples and three SAHA-treated samples (100 μg, each) were labeled by TMT 6-plex reagents (Thermo Fisher Scientific) with TMT126, TMT127, TMT128, TMT129, TMT130, and TMT131, respectively. Each sample was combined with its respective 6-plex TMT reagent and incubated for 1 h at room temperature. Then, hydroxylamine was added to the sample and incubated for 15 min to quench the reaction. Equal amounts of each TMT-labeled sample were combined in new microcentrifuge tubes and lyophilized in a SpeedVac concentrator.

### Phosphorylated and Acetylated Peptide Enrichment

Phosphopeptide enrichment was performed as described by Larsen et al. Briefly, lyophilized peptides were re-suspended in DHB buffer [3% w/v DHB, 80% v/v ACN, and 0.1% v/v trifluoroacetic acid (TFA)]. Then, titanium dioxide beads (GL Sciences, Japan) were added, and the mixture was agitated for 40 min. TiO_2_ beads were recovered by centrifugation and washed three times with washing buffer I (30% ACN and 3% TFA) and three times with washing buffer II (80% ACN and 0.3% TFA). Last, the phosphopeptides were eluted with elution buffer (5% NH4OH/50% ACN), followed by lyophilization and MS analysis.

Prior to acetylated peptide enrichment, anti-lysine acetylation (Kac) antibody beads (PTM Biolabs, Inc., Hangzhou) were washed twice with ice-cold PBS. To enrich Kac peptides, 5 mg tryptic peptides of Kac were dissolved in NETN buffer (100 mM NaCl, 1 mM EDTA, 50 mM Tris–HCl, and 0.5% NP-40, pH 8.0) and incubated with pre-washed antibody beads (Catalog No. PTM-104, PTM Biolabs, Inc., Hangzhou) in a ratio of 15 ml beads/mg protein at 4°C overnight with gentle shaking. The beads were washed four times with NETN buffer and twice with ddH_2_O. The bound peptides were eluted from the beads with 0.1% TFA. The eluted peptides were collected and vacuum-dried followed by LC-MS/MS analysis.

### LC−ESI−MS/MS Analysis by Q-Extractive MS

Peptides were dissolved in solvent A (0.1% FA), and loaded onto a Thermo Scientific EASY column (C18 column, 5 μ m, 100 μ m × 2 cm, Thermo Scientific). Peptide separation was performed using a reversed-phase analytical column (C18 column, 75 μ m × 250 mm, three μ m, Thermo Scientific). The gradient was comprised of an increase from 0% to 55% solvent B (0.1% FA in 98% ACN) for 220 min, 55%–100% for 8 min, and then holding at 100% for the last 12 min, at a constant flow rate of 250 nl/min on an EASY-nLC 1000 UPLC system. The eluted peptides were analyzed using the Q Exactive™ hybrid quadrupole-Orbitrap mass spectrometer (Thermo Scientific). A data-dependent procedure was one MS scan (m/z range of 350–1800) followed by 10 MS/MS scans for the top 20 precursor ions. Dynamic exclusion was enabled with an exclusion duration of 30 s. Automatic gain control (AGC) was set at 3e6 to prevent overfilling of the ion trap. The peptides were detected in the Orbitrap at a resolution of 70,000. Peptides were selected for MS/MS using the NCE setting as 29, and ion fragments were detected at a resolution of 17,500.

### MS Data Analysis

MS/MS spectra were searched using Mascot 2.2 (Matrix Science) embedded in Proteome Discoverer 1.4 against the UniProt human FASTA (released on 5/5/2018). For protein identification, the following options were used: peptide mass tolerance, 20 ppm; MS/MS tolerance, 0.1 Da; enzyme, trypsin; missed cleavage, two; fixed modifications, carbamidomethyl (C); variable modifications, TMT 6-plex (N-term), TMT 6-plex (K), oxidation (M), and phosphorylation (S/T/Y); and false discovery rate (FDR) ≤ 0.01. Proteome Discoverer 1.4 was used to extract the peak intensity of each expected TMT reporter ion from the fragmentation spectrum. Only spectra in which all quant channels are present were used for quantification. The score threshold for peptide identification was set at 1% FDR and with a phosphorylated and acetylated site probability cutoff of 0.75. Student’s t-test was used to evaluate the statistical significance, and FDR was calculated. The criteria for significant abundance changes were the abundance ratios ≥1.2 and the *p*-value ≤ 0.05.

### Bioinformatic Analysis

Gene Ontology (GO) term association and enrichment analysis were performed using the Database for Annotation, Visualization, and Integrated Discovery (DAVID). The Encyclopedia of Genes and Genomes (KEGG) database was used to identify enriched pathways by the Functional Annotation tool of DAVID against the background of *Homo sapiens*. The InterPro database was researched using the Functional Annotation tool of DAVID against the background of *Homo sapiens*. A manually curated CORUM protein complex database for human was used for protein complex analysis. To construct a protein–protein interaction network, the STRING database system was used. Functional protein interaction networks were visualized using Cytoscape. When performing the bioinformatic analysis, the corrected *p*-value of 0.05 was considered significant. Also, all the detailed description of bioinformatic analysis is listed in Supplementary Information.

## Results

### Suberoylanilide Hydroxamic Acid Treatment Changes the Proteome Profile in Nasopharyngeal Carcinoma Cells

It has been reported that SAHA altered the global proteome in several kinds of cancers to deploy anti-tumor activity (Wu et al., 2013; Garmpis et al., 2017). Since the 5–8 F cell line is one of the representative models used to study the molecular events of NPC metastasis, we chose it to perform multiple omics toward SAHA treatment. In our previous study, the maximum H3Kac signal was detected at 6 μM and 24 h of SAHA treatment. Since the cell viability was still nearly 80% when treated at 6 μM for 24 h, this condition was applied for all the following experiments in NPC cells (Huang et al., 2020). In the current study, 6,391 proteins were quantified in the 5–8 F cell line upon SAHA treatment. Among the 6,391 quantified proteins, 454 were up-regulated and 217 were down-regulated with 1.2 change folds.

**FIGURE 1:**
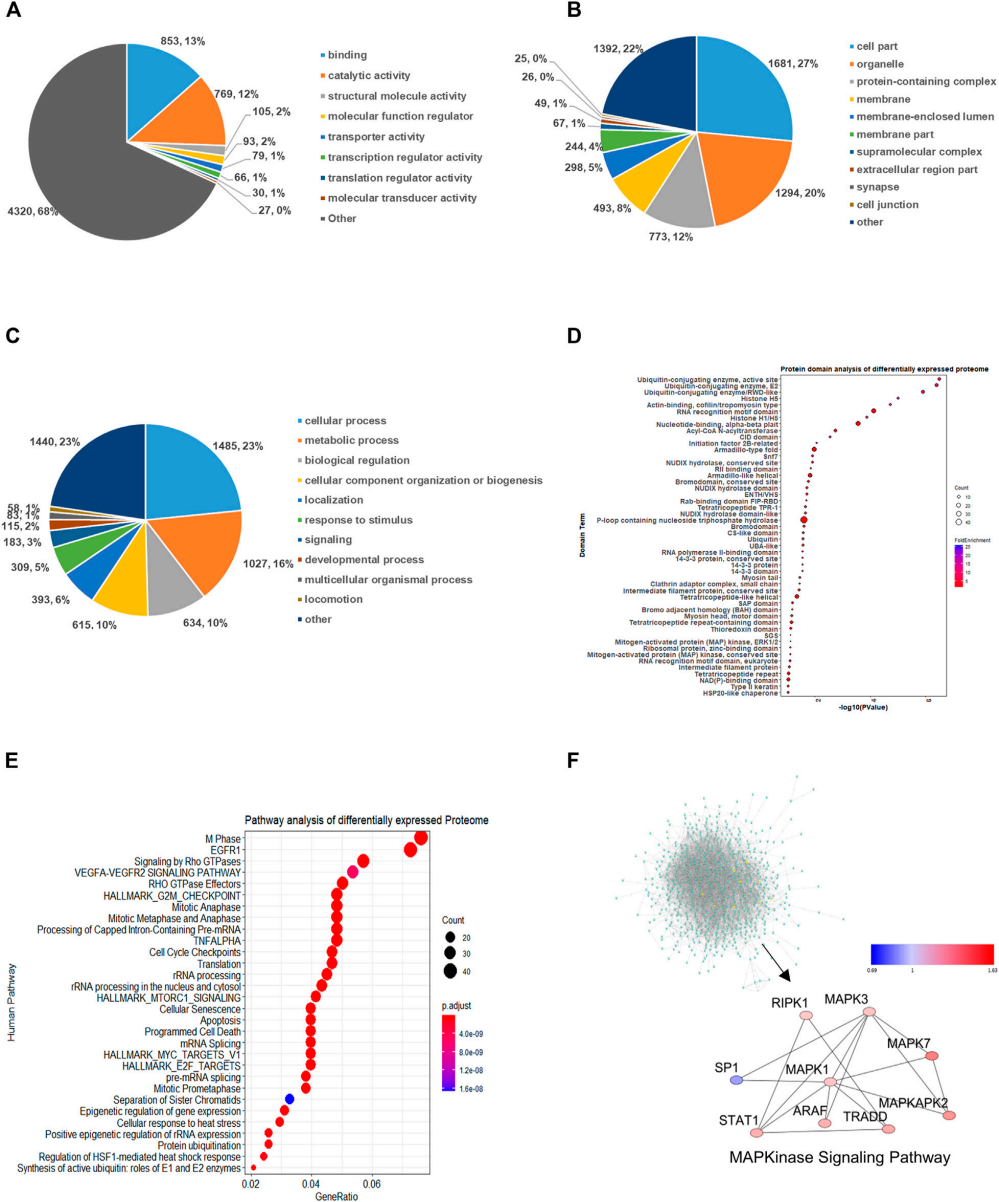
Functional enrichment cluster analysis of quantified global proteome. **(A)** Molecular function. **(B)** Cellular compartment. **(C)** Biological process. **(D)** Protein domain analysis. **(E)** KEGG pathway enrichment analysis of differentially expressed proteins. **(F)** Protein–protein interaction network of proteome. The global view and representative MAPK pathway are displayed.

To illustrate the functions of the differentially expressed proteins (DEPs), comprehensive bioinformatics analyses were performed via Gene Ontology (GO) analysis, protein domain analysis, and Kyoto Encyclopedia of Genes and Genomes (KEGG) pathway analysis (Figures 1A–E). For the molecular function (MF) category, the DEPs were highly enriched in binding, catalytic activity, and structural molecule activity (Figure 1A). The analysis of the cellular compartment (CC) displayed those proteins that are involved in cell part, organelle, and protein-containing complex and were enriched toward SAHA treatment (Figure 1B). Biological process (BP) analysis revealed that DEPs were mainly associated with cellular process, metabolic process, biological process, and cellular component organization (Figure 1C).

The protein-specific structural domain is one of its major functional characteristics. Consequently, we investigated the enriched domains of quantified proteins upon SAHA induction (Figure 1D). We found that those domains related to ubiquitin-conjugating enzyme active site, nucleotide binding, histone H1/H5 domain, and RNA recognition motif domain were highly enriched. To further explore the relevant pathways regulated by SAHA treatment, KEGG pathway analysis was performed. As shown in Figure 1E, spliceosome, endocytosis, and ubiquitin-mediated proteolysis pathways were the three most robustly enriched ones in response to SAHA stimulation, indicating the function of SAHA in regulating these signaling pathways.

Finally, we established the protein–protein interaction network via the STING database and visualized by Cytoscape software (Figure 1F). The global network of PPI and the highly enriched MAPKinase signaling pathway were presented. MAPK signaling, which regulates gene expression, cellular growth, and survival, plays a vital role in tumorigenesis. It has been demonstrated that its dysregulation may lead to abnormal cell proliferation and resistance to apoptosis (Bruzzese et al., 2011; Citro et al., 2019). This result indicated that the MAPK signaling pathway might be a potential target of SAHA in NPC cells.

### Suberoylanilide Hydroxamic Acid Treatment Changes the Acetylome Profile in Nasopharyngeal Carcinoma Cells

Given that SAHA is a pan HDAC inhibitor, we suspected that it altered protein acetylation in NPC cells. To this end, we performed quantitative acetylomics toward SAHA treatment in 5–8 F cells by combination of TMT labeling, antibody enrichment of acetylation, and LC-MS/MS analysis. Altogether, 441 lysine acetylation sites located in 298 proteins were identified, of which 333 sites located in 228 proteins were quantified. Within these quantified acetylation sites, 32 sites distributed on 26 proteins were upregulated and 47 sites distributed on 45 proteins were downregulated upon SAHA treatment (fold change >1.2 or < -1.2). Also, top ten acetylated sites and corresponding proteins upon SAHA stimulation were concluded (Table 1).

**TABLE 1:** Top ten acetylated sites and corresponding proteins with the highest fold changes in acetylome upon SAHA treatment in 5–8 F cells.

Symbol	Position	Fold change	Acetylated Probability
PHIP	1399	26.26	AYTPSK(1)R
PLXNB2	941	25.59	VTK (1)FGAQLQCVTGPQATR
SLC25A5	105	24.57	QIFLGGVDK (1)R
ALDOA	147	23.78	DGADFAK (1)WR
CREBBP	1595	23.2	TNK(1)NKSSISR
CREBBP	1597	23.2	TNKNK(1)SSISR
NOLC1	251	23.15	KQVVAK (1)APVK
CTTN	272	22.99	TGFGGK (1)FGVQSER
MACROH2A1	142	22.7	SPSQK (0.964)KPVSK
CTTN	235	22.4	GFGGK (1)FGVQTDR
ACTN4	497	−21.86	CQK (1)ICDQWDALGSLTHSR
AHNAK	4239	−21.9	VDIDVPDVNIEGPDAK (1)LK
KMT2A	1130	−21.98	SSIAGSEDAEPLAPPIK(1)PIKPVTR
AHNAK	1305	−22.07	VDVEVPDVSLEGPEGK (1)LK
SF3A1	486	−22.32	RTDIFGVEETAIGK (1)K
MRPL47	144	−22.36	VVDSMDALDK (1)VVQER
CLTA	242	−22.37	SVLISLK (1)QAPLVH
HSPD1	473	−22.95	TLK (1)IPAMTIAK
SET	7	−23.9	SAPAAK (1)VSKK
EAF1	150	−25.11	APTK (1)PPVGPK

**FIGURE 2:**
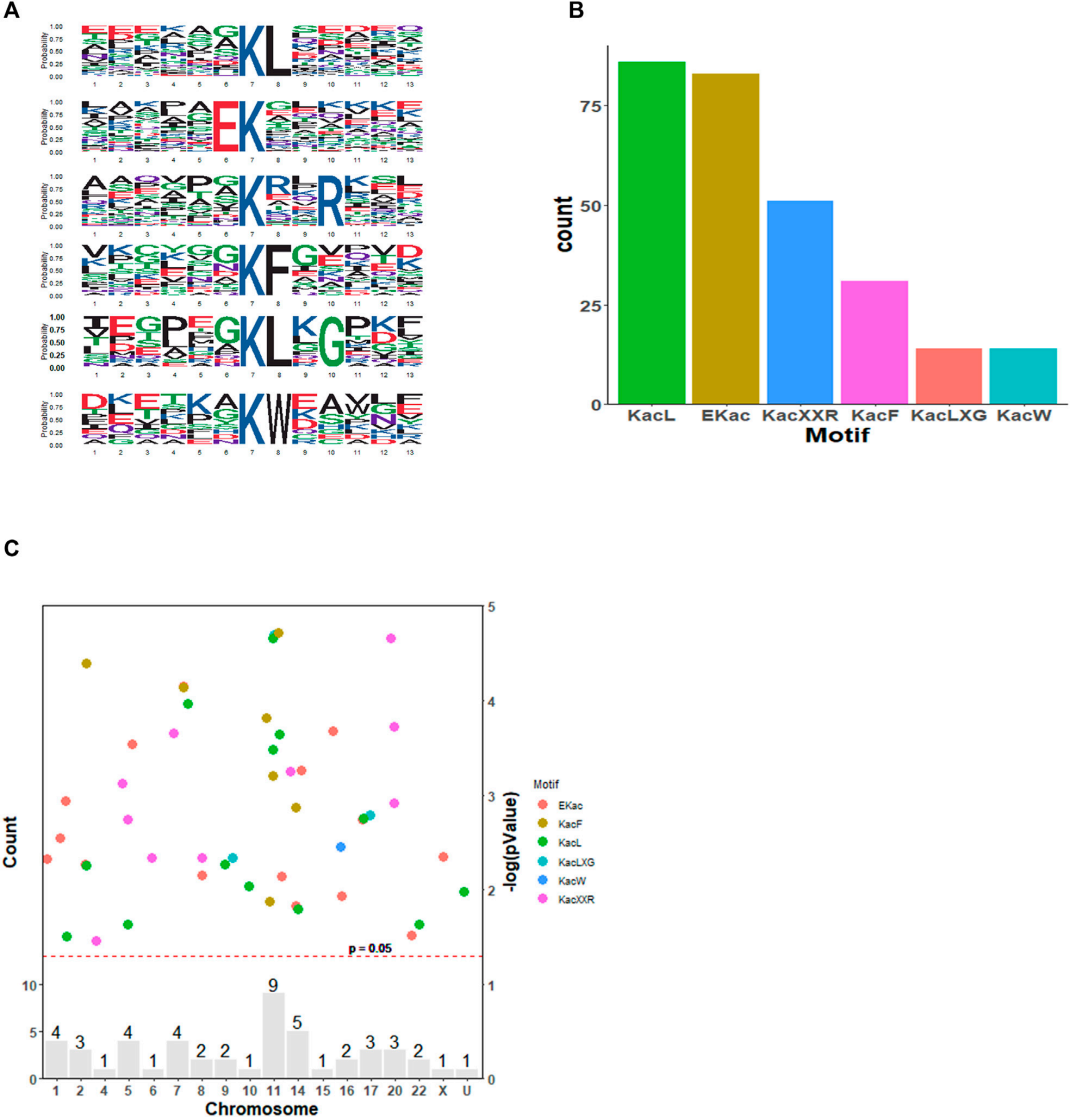
Motif analysis of the identified acetylation peptides in SAHA-treated cells. **(A)** Sequence logo of acetylated motifs. **(B)** Number of identified peptides containing acetylated lysine in each motif. **(C)** Distribution of acetylated proteins across chromosomes. The red dashed line corresponds to *p = 0.05*, and the peptide sites (dots)above this threshold are shown in different colors according to their motifs. The bars indicate the total number of acetylated sites in each chromosome. U, undetermined.

To understand the features of these acetylated proteins, integrated bioinformatics analyses in combination with motif analysis, GO category analysis, KEGG pathway, and protein–protein interaction analysis were conducted. As shown in Figure 2A, we studied the amino acids located around acetylated sites via a motif analysis approach. In total, six motifs were robustly enriched: KacL, EKac, Kac**R, KacF, KacL, and KacW (Kac refers to acetylated lysine, and * refers to the random amino acid site). These six motifs differed in abundance and KacL, EKac, and Kac**R comprised approximately 66% of all quantified peptides (Figure 2B). SAHA altered histone acetylation, which had a great impact on chromatin remodeling and epigenetics (Kim and Kaang, 2017). Accordingly, distribution of acetylated proteins across chromosomes was analyzed, which demonstrated that these proteins were mainly located at No.11 and No.14 chromatins (Figure 2C). The results suggested that SAHA preferred to acetylate specific motifs of proteins distributed at certain chromatins in NPC cells.

**FIGURE 3:**
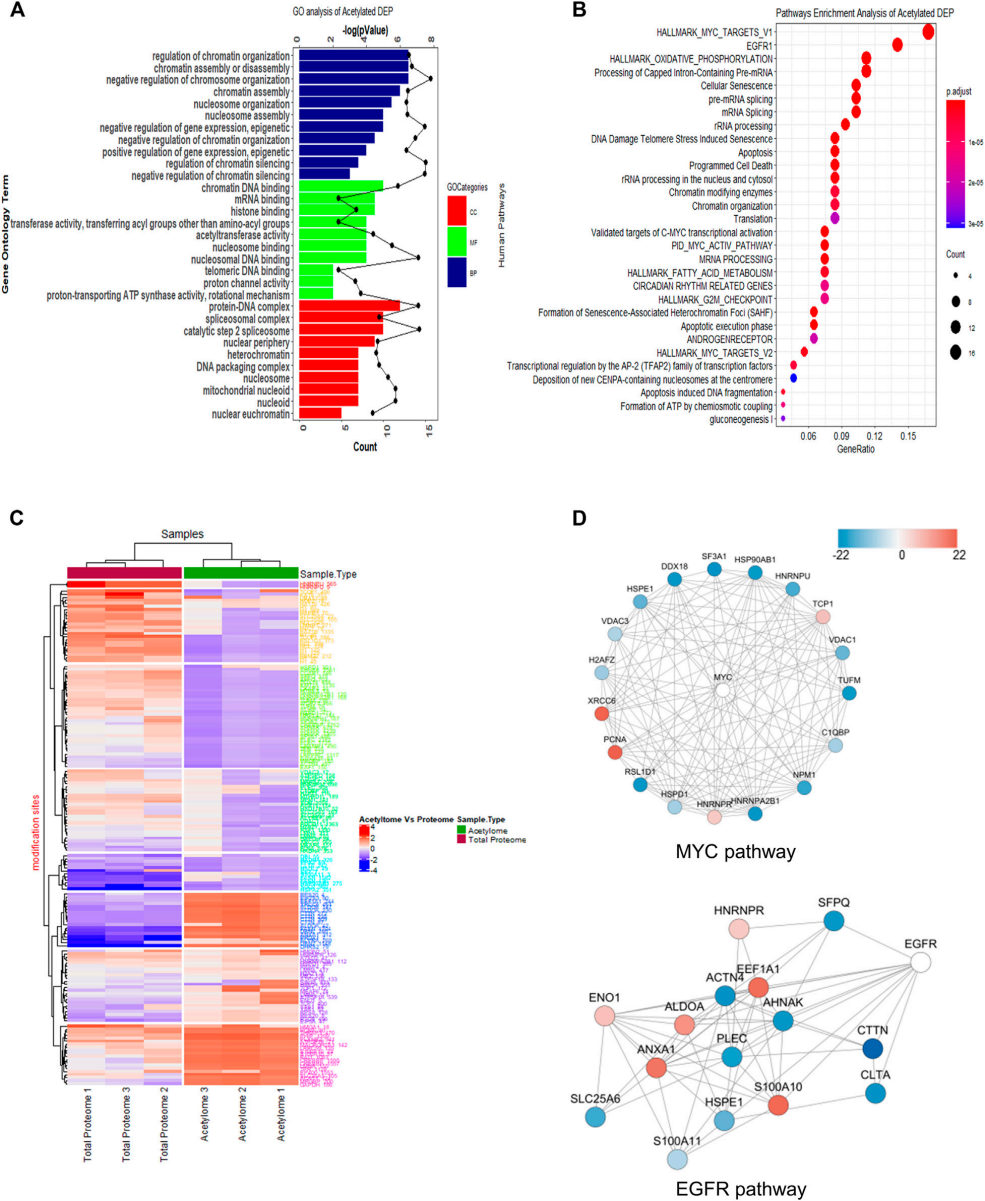
Functional enrichment analysis of quantified acetylated proteins. **(A)** GO enrichment analysis. **(B)** KEGG pathway analysis. **(C)** Heatmap diagram of a two-way hierarchical clustering. The red and blue colors indicate the expression level of proteins in terms of normalized ratio. Upper color labeling shows total proteome samples in red and acetylome in green. The distance of hierarchical clustering was measured using the Euclidean method. **(D)** Protein–protein interaction network of acetylome clustered in the representative MYC and EGFR pathway.

When the GO database was applied to analyze these acetylated proteins, we found that the proteins mainly participated in regulation of RNA splicing process, ribonucleotide triphosphate metabolic process, and purine ribonucleotide triphosphate metabolic process with biological process (BP) category analysis (Figure 3A, top panel). In terms of the molecular function (MF) category, chromatin DNA binding, nuclear receptor transcription coactivator activity, and nucleosome binding were the top three items (Figure 3A, middle panel). Also, as shown in cellular component (CC) analysis, these acetylated DEPs mainly involved in spliceosome complex, catalytic step 2 spliceosome, and mitochondrial protein complex (Figure 3A, bottom panel). These results revealed that SAHA may regulate RNA splicing process, metabolic-related process, and chromatin DNA-binding activity to play anti-tumor activities in NPC cells.

Next, the KEGG pathway was conducted to have a look into the associated cellular signaling toward SAHA treatment. Totally, based on the criteria, i.e., *p* < 0.05 and FDR <0.05, the DEPs took part in 30 prevalent cellular signaling pathways (Figure 3B). Among them, MYC, EGFR, mRNA splicing, G2M checkpoint, oxidation phosphorylation, and TCA cycle were mainly enriched pathways, which were closely associated with cell proliferation, cellular metabolism, and cell cycle processes. It has been well characterized that these notable processes are hallmarks of cancer (Hanahan and Weinberg, 2011). These results hinted that SAHA may modulate these tumor-associated signaling pathways via protein acetylation to display treatment functions in NPC cells.

In Figure 3C, we clustered the differentially expressed acetylated proteins via two-way hierarchical clustering, which clearly displayed the patterns of the acetylated and global proteins in response to SAHA treatment. Taking advantage of the STING database, the PPI network was studied. Representative protein–protein interactions of MYC and EGFR signaling pathways are shown in Figure 3D. In the MYC signaling pathway, there were 14 sites that exhibited a decrease at the acetylation level upon SAHA treatment, while four sites were increased. For the EGFR signaling pathway, there were 15 proteins quantified to be acetylated, six of which were upregulated and nine were downregulated (Figure 3D). Taken together, the results retrieved from the acetylome data suggested that SAHA acetylated proteins mainly located at specific chromatins, further regulated downstream genes and cellular processes to exert anti-tumor effects in NPC cells.

### Crosstalk Between Global Proteome and Acetylome in Nasopharyngeal Carcinoma Cells

**FIGURE 4:**
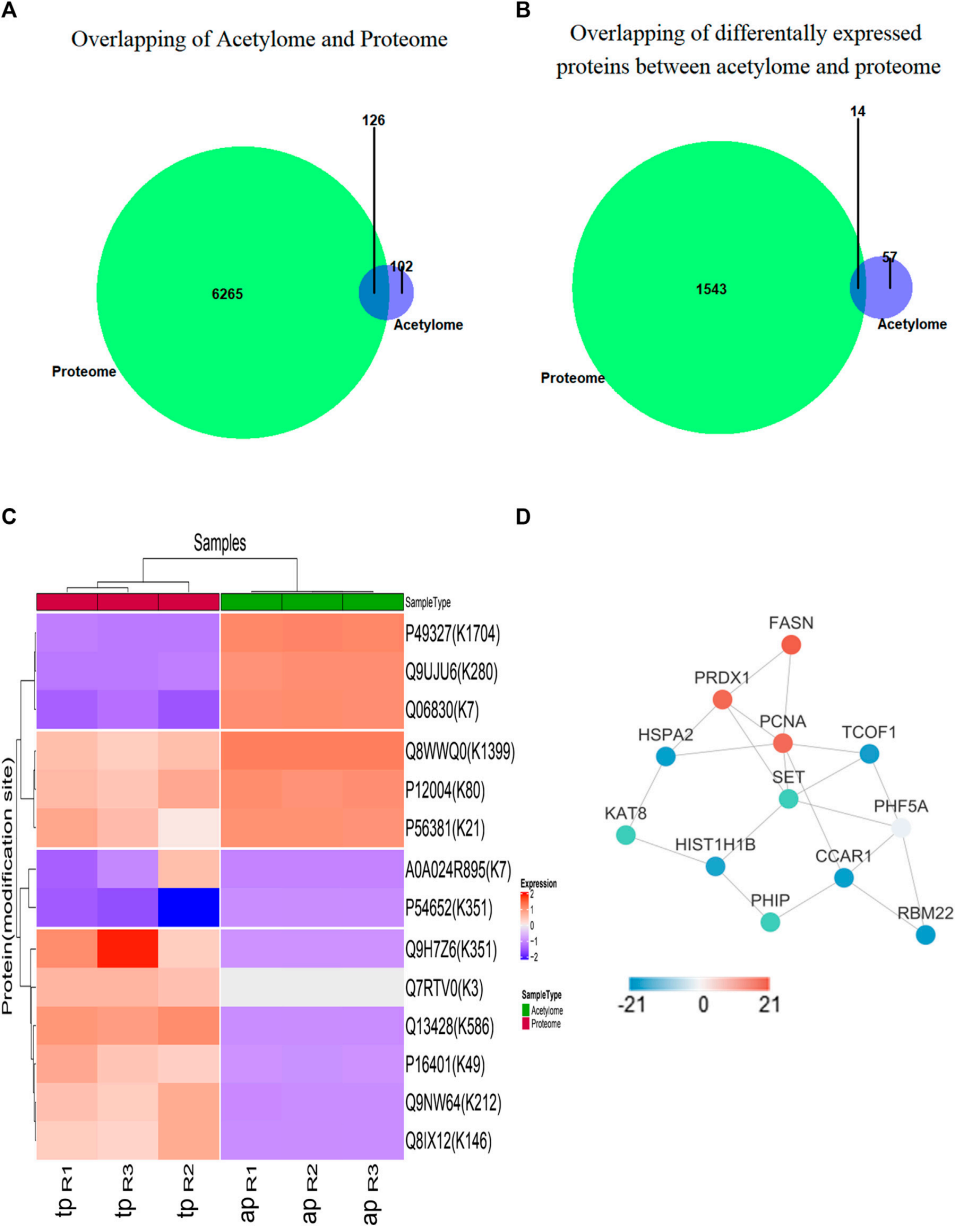
Functional analysis between proteome and acetylome. **(A)** Overlap of total proteins between acetylome and proteome. **(B)** Overlap of differentially expressed proteins between acetylome and proteome. **(C)** Heatmap diagram of a two-way hierarchical clustering. Diagram consists of the 14 most differentially expressed proteins in the global proteome and acetylome. The red and blue colors indicate the expression level of proteins in terms of normalized ratio. Upper color labeling shows total proteome samples in red and acetylome in green. The distance of hierarchical clustering was measured using the Euclidean method. **(D)** Protein–protein interaction network of differentially expressed proteins between acetylome and proteome.

According to the whole proteome and acetylome data from 5–8 F cells upon SAHA treatment, the crosstalk between these two modifications was analyzed. In total, 126 proteins were quantified by proteome and acetylome, including 14 DEPs (Figures 4A, B). Subsequently, these 14 DEPs were selected for two-way hierarchical clustering analysis (Figure 4C). The protein–protein interaction analysis was studied to unveil the functional relationship between these two profiles, and the representative interacted proteins are displayed in Figure 4D. Together, these analyses indicated a complex relationship between proteome and acetylome, which synergistically determined the fate of SAHA in NPC cells.

### Suberoylanilide Hydroxamic Acid Treatment Changes the Phosphoproteome Profile in Nasopharyngeal Carcinoma Cells

Phosphorylation is one of the most important PTMs and is believed to take part in the processes of cancer progression (Krueger and Srivastava, 2006). In a previous study, we demonstrated that SAHA regulated the phosphorylation of p53 and Rb1 in NPC cells; hence, protein phosphoproteome toward SAHA stimulation was investigated here.

**FIGURE 5:**
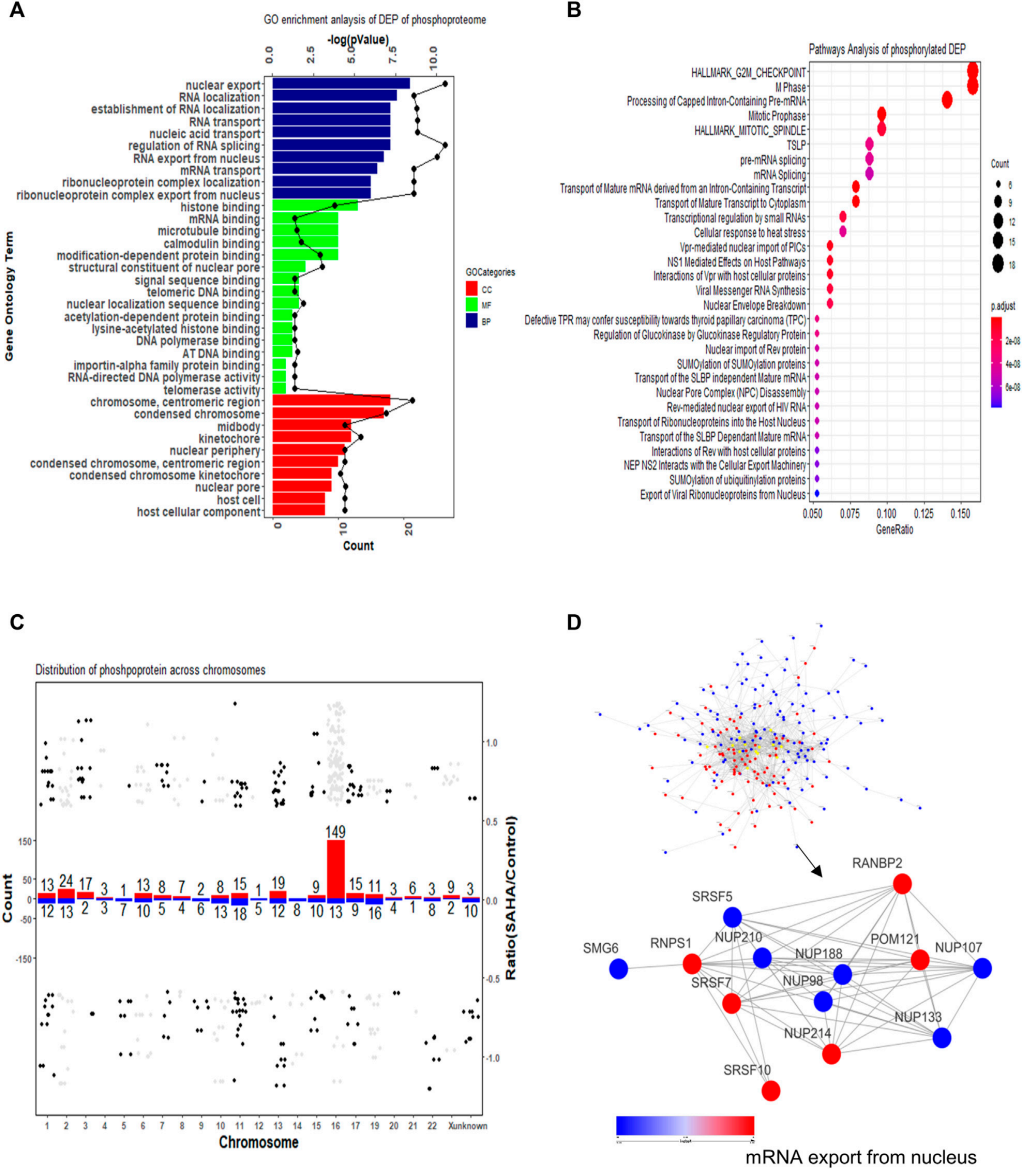
Functional enrichment cluster analysis of quantified phosphorylated proteins. **(A)** GO enrichment analysis. **(B)** KEGG pathway analysis. **(C)** Distribution of phosphorylated proteins across chromosomes. The number above each bar indicated the total number of phosphorylated proteins in each chromosome. The red bar refers to upregulated proteins; the blue bar refers to downregulated proteins. **(D)** Protein–protein interaction network of phosphoproteome. Both global network and representative mRNA export processes are displayed.

Classification and enrichment analysis based on GO revealed that the DEPs in phosphoproteome were mainly associated with RNA-related processes in the biological process (BP) category, such as RNA localization process, nucleobase-containing compound transport process, establishment of the RNA localization process, and RNA/mRNA transport process. The cellular component (CC) category revealed that DEPs were primarily in chromosome, including chromosome centromeric region, condensed chromosome, and heterochromatin. For the molecular functional (MF) category, mRNA binding, histone binding, modification-dependent protein binding, and RNA polymerase binding were highly enriched (Figure 5A).

To further study the related cellular signaling of phosphorylated DEPs, we conducted signaling analysis via the KEGG database. In total, 30 significant cellular pathways were detected under the criteria *p* < 0.05 and FDR <0.05 (Figure 5B). It showed that cell cycle and mRNA splicing-related signaling pathways were robustly affected, which included G2M checkpoint pathway, M phase, mitotic spindle pathway, and pre-mRNA/mRNA splicing pathway. Intriguingly, SUMOylation-related signaling was enriched in the SAHA-induced phosphoproteome profile, which is consistent with the previous study that SAHA regulated protein sumoylation to implement biological functions.

Then, distribution of phosphorylated proteins across chromosome was also studied. The result presented that enhanced phosphorylated proteins were mainly gathered in No.16 and No.2 chromatins, while decreased phosphorylated proteins were mostly located at No.11 and No.19 chromatins (Figure 5C). The protein–protein interaction network of phosphorylated proteins was also established. The overview network and the representative mRNA export process are presented in Figure 5D. The result uncovered that SAHA boosted some proteins’ phosphorylation levels (RANBP2, POM121, NUP214, SRSF10, and RNPS1) while reduced the phosphorylation state of others (SRSF5, NUP210, NUP188, NUP98, NUP107, NUP133, and SMG6) (Figure 5D). Taken together, our results indicated that SAHA may manipulate the phosphorylation of some critical molecules to regulate pivotal signaling pathways, leading to the therapeutic roles in NPC cells.

### Crosstalk Between Phosphoproteome and Acetylome in Nasopharyngeal Carcinoma Cells

**FIGURE 6:**
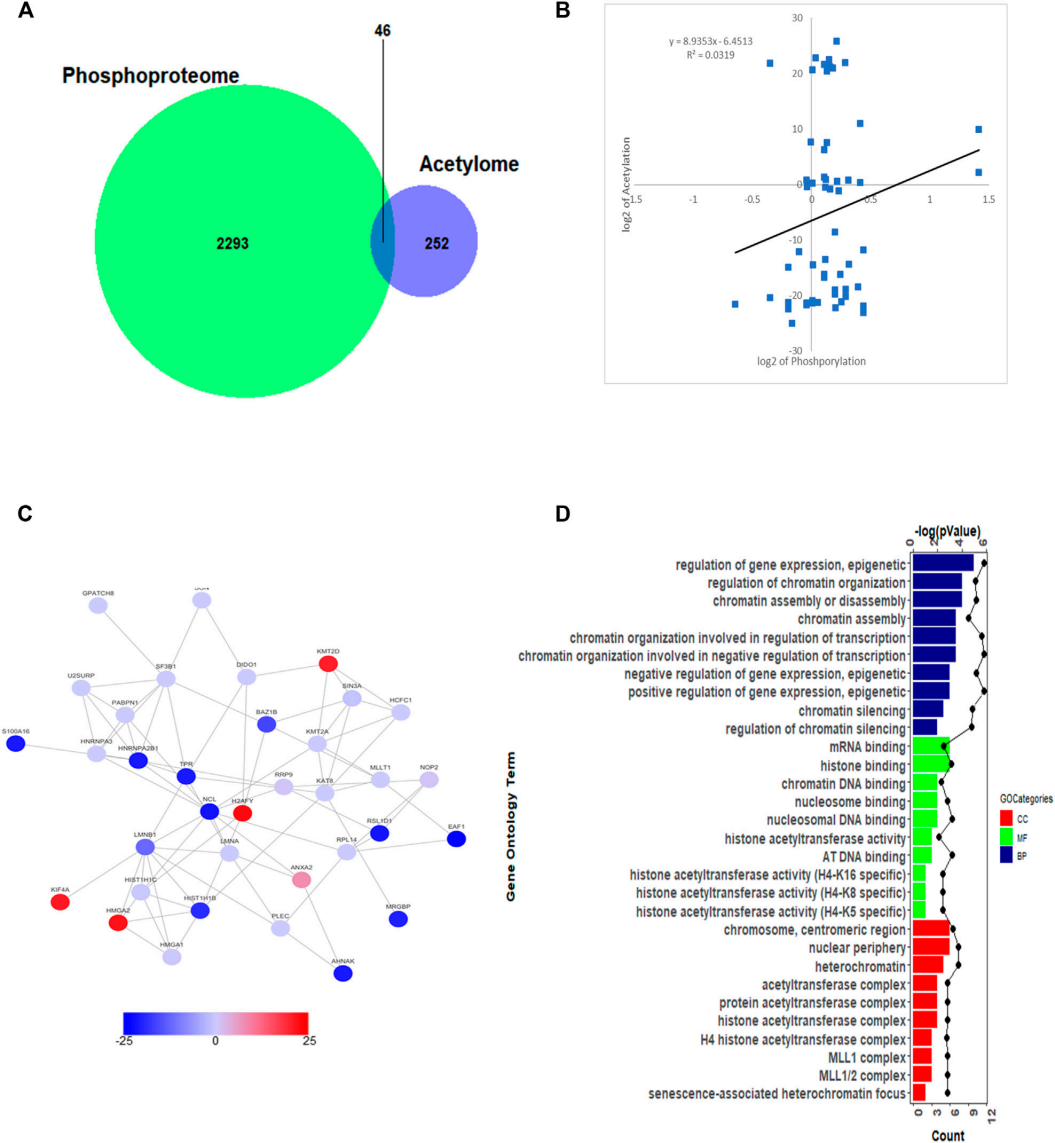
Crosstalk analysis between phosphoproteome and acetylome. **(A)** Overlap of total proteins between acetylome and phosphoproteome. **(B)** Correlation between acetylome and phosphoproteome. **(C)** Protein–protein interaction network between acetylome and phosphoproteome. **(D)** GO enrichment analysis between acetylome and phosphoproteome.

It was reported that each protein modification can crosstalk with one or more other modifications to affect cellular functions (Hunter, 2007). As the most prominent two PTMs, phosphorylation and acetylation were demonstrated that linked closely with each other. To this point, we compared the acetylation and phosphorylation data from 5–8 F cells treated with SAHA, and 46 proteins were identified, which were both acetylated and phosphorylated (Figure 6A). From the scatterplot, the connection ratio is 0.0319, which meant the two protein modifications were not directly linked overall (R^2^ = 0.0319, Figure 6B).

To better understand the relationship between these two protein modifications, we conducted the interaction network of proteins. The overall diagram of protein interaction is presented in Figure 6C. Then, through clustering these proteins, we found out that both acetylated and phosphorylated proteins take part in regulating prevalent biological processes, such as gene expression, epigenetics, chromatin organization, chromatin assembly, and disassembly processes (Figure 6D). For molecular functional (MF) analysis, mRNA binding, histone binding, chromatin DNA binding, and histone acetyltransferase activity were the mostly clustered items. Subsequently, the proteins detected were mainly located in chromosome, nuclear periphery, heterochromatin, and acetyltransferase complex (Figure 6D). The compressive analyses suggested that SAHA mediated both protein acetylation and phosphorylation to regulate cellular processes, further deployed inhibitory roles in NPC cells.

### Validating the Phosphorylated and Acetylated Proteins by Suberoylanilide Hydroxamic Acid Treatment in Nasopharyngeal Carcinoma Cells

**FIGURE 7:**
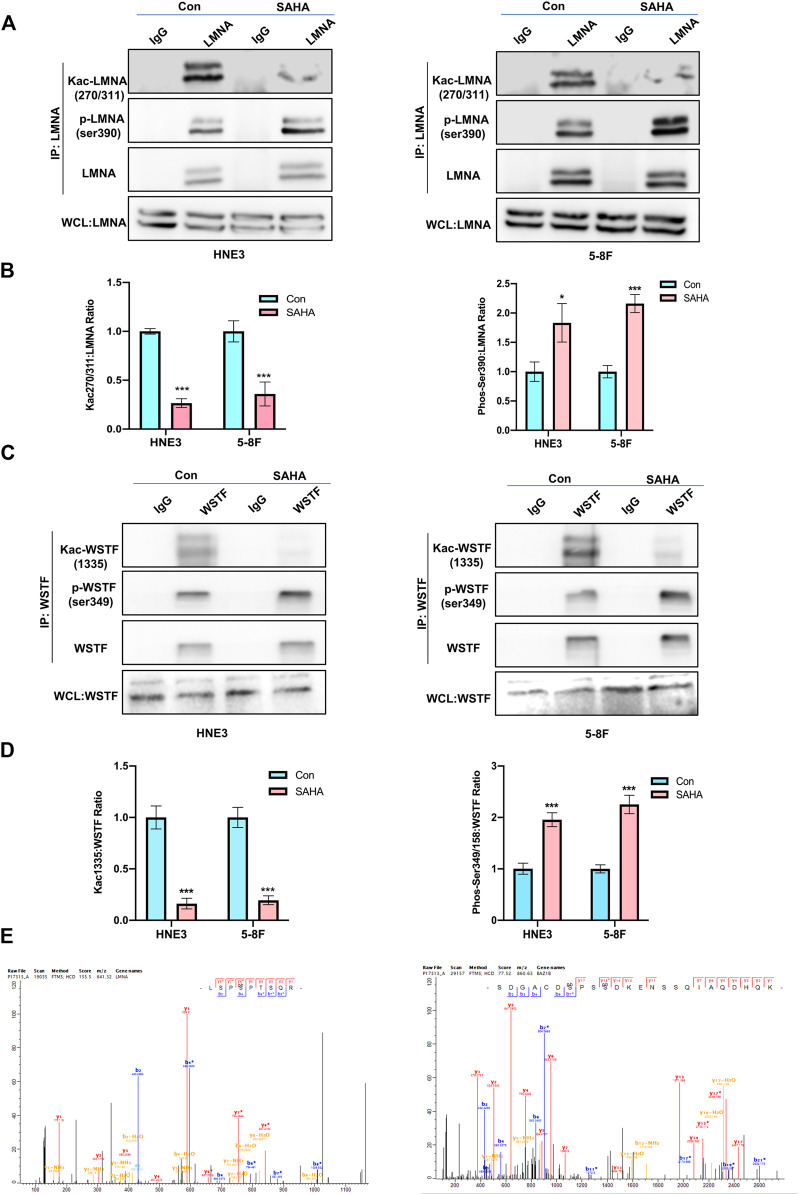
Validation of the key phosphorylated and acetylated proteins by SAHA. **(A**,**B)** Immunoblot analysis of LMNA phosphorylation and acetylation in NPC cells with or without SAHA treatment. NPC cells (HNE3 and 5–8 F) were treated with or without SAHA (6 μM) for 24 h; WCLs were precipitated with anti-LMNA or control antibodies; precipitated proteins and WCLs were analyzed by immunoblotting with indicated antibodies. Protein levels of LMNA are shown in **(A)**, and the statistical analysis of all samples is shown in **(B)**. * denotes *p* < 0.05, ***denotes *p* < 0.001. Error bars represent ± SD of triplicate experiments. **(C**,**D)** Immunoblot analysis of WSTF phosphorylation and acetylation in NPC cells with or without SAHA treatment. NPC cells (HNE3 and 5–8 F) were treated with or without SAHA (6 μM) for 24 h; WCLs were precipitated with anti-WSTF or control antibodies; precipitated proteins and WCLs were analyzed by immunoblotting with indicated antibodies. Protein levels of WSTF are shown in **(C)**, and the statistical analysis of all samples is shown in **(D)**. ***denotes *p* < 0.001. Error bars represent ± SD of triplicate experiments. **(E)** Mapping and quantification of LMNA and WSTF phosphopeptides. Representative MS2 spectrum derived from LMNA and WSTF containing serine phosphorylation sites.

Based on raw quantitative MS data, we listed top ten sites that underwent both phosphorylation and acetylation and corresponding proteins upon SAHA stimulation in NPC cells (Table 2). Williams syndrome transcription factor (WSTF) and lamin A protein (LMNA) were the top proteins in our analysis, both of which had been reported to involve in tumorigenesis and development. Given that, we examined the phosphorylation and acetylation levels of the two proteins to further confirm our results. Using Western blotting analysis, we showed that SAHA treatment induced the accelerated phosphorylation signals of LMNA at the Ser390 site, while its acetylation status at K270/311 was declined in different NPC cell lines, including 5–8 F and HNE3 cells (Figures 7A, B). Similar results were observed in WSTF protein, as shown in Figure 7C, and demonstrated that the phosphorylation signals of Ser349/158 were improved and acetylation levels of K1335 were reduced in SAHA-treated cells (Figures 7C, D), which were consistent with the quantitative MS analysis. The representative MS2 spectrum of WSTF and LMNA proteins are presented in Figure 7E. Taken together, these results demonstrated that SAHA may elicit anti-tumor activity in NPC cells via regulating both phosphorylation and acetylation of WSTF and LMNA at specific residues.

**TABLE 2:** Top ten sites that underwent both acetylation and phosphorylation with fold changes and corresponding proteins upon SAHA treatment in 5–8 F cells.

Accession	Symbol	Acetylated site	Fold change	Phosphorylated site	Fold change
P18583	SON	2055	−25.76	S2020/S2022	2.53
P18583	SON	2055	−25.76	S2029/S2031	1.56
P02545	LMNA	270	−23.85	S390	1.42
Q9UIG0	WSTF	1335	−23.60	S349	1.75
Q9UIG0	WSTF	1335	−23.60	S158	1.68
P02545	LMNA	311	−23.33	S390	1.42
Q09666	AHNAK	2561	−21.69	S5448	1.29
Q09666	AHNAK	2561	−21.69	S5863	1.29
Q96T23	RSF1	1390	−21.24	S1277/T1278	1.51
Q96T23	RSF1	1390	−21.24	T1305	1.29

## Discussion

NPC is a malignant tumor of the head and neck with poor prognosis. Although great progress has been made in treatment, especially in radiotherapy, the 5-year overall survival rate is still low due to the recurrence and metastasis (Cao et al., 2011). It is urgent to develop predictive prevention and personalized medicine for NPC patients (Cheng and Zhan, 2017). SAHA has been demonstrated to implement anti-tumor effect in various cancers, including NPC (Huang et al., 2020). However, the underlying mechanisms of its anti-tumor activity are still not well clear. In the current work, we utilized a TMT-based quantitative proteomic method, which integrated the data from proteome, acetylome, and phosphoproteome, to study the anti-tumor effect of SAHA in NPC cells.

Since SAHA belongs to the deacetylase inhibitor family, we performed acetylome in 5–8 F cells. The quantitative acetylation data revealed that 333 acetylated sites in 228 proteins were detected following SAHA treatment. From these acetylated sites, 32 sites of 26 proteins were upregulated and 47 sites of 45 proteins were downregulated. These apparently changed proteins that took part in a variety of biological functions, of which MYC and EGFR pathways ranked the top two enriched pathways (Figure 3B).

MYC is one of the most highly amplified oncogenes, whose deregulation is commonly found on the path to cancer, including myeloma, head and neck tumors, lymphoma, and breast cancer (Dang, 2012). In addition, MYC was shown to participate in several types of small molecular inhibitor resistance. It demonstrated that K-Ras conferred SAHA resistance by upregulating HDAC6 and MYC expression in colon cancer cells (Wang et al., 2016a). Wajana L et al reported that MYC controlled the sensitivity of gastric cancer upon HDAC inhibitors via directly regulating MCL1 and eIF4E gene transcription (Labisso et al., 2012). Other groups also elucidated that MYC inhibitors and HDAC inhibitors, such as SAHA, may have cross effects to fight against cancers. A combination of these two kinds of inhibitors might be a promising therapeutic choice for cancer patients (Allen-Petersen and Sears, 2019). Consistent with these research studies, our present results uncovered that SAHA treatment significantly altered the acetylation level of the MYC pathway in 5–8 F cells, which might be one of the principles of SAHA to play roles in NPC cells. Moreover, our study provided useful resources to further investigate the cross effects between HDAC inhibitors and MYC inhibitors.

The epidermal growth factor receptor (EGFR) exerts critical functions in epithelial cell physiology, which belongs to the ErbB family of receptor tyrosine kinases (RTKs) (Schlessinger, 2014). Its deregulation, such as mutation and/or overexpression, was reported in different types of human cancers, including head and neck cancer. Thus, EGFR signaling is a promising therapeutic target. He et al (2019) revealed that TSA, an HDAC inhibitor, decreased the EGFR-Arf1 signaling to inhibit cell migration and invasion in SCCHN. Citro et al (2019) demonstrated that SAHA, in combination with gefitinib, displayed synergistic anti-tumor activities in SCCHN cell lines via disturbing EGFR receptor expression. These reports supported our finding that SAHA treatment may alter critical molecules’ acetylation in EGFR signaling, which contributed to the anti-tumor effect of SAHA in NPC cells.

Protein functions are mediated by a number of PTMs, which subsequently manipulate key cellular processes (Swaney et al., 2013). Phosphorylation and acetylation are the two most prevalent PTMs in the eukaryotic proteome (Kim et al., 2006). In the present study, we performed the global proteome, acetylation, and phosphorylation in 5–8 F cells following SAHA stimulation. Our results showed that in addition to the well-established effects on protein acetylation, SAHA also regulated global proteome and phosphorylation. Considering the growing evidence that indicated the link between acetylation and phosphorylation, it is reasonable to analyze the crosstalk among global proteome, acetylation, and phosphorylation toward SAHA stimulation in NPC cells. According to our data, SAHA treatment directly altered protein acetylation and phosphorylation, and the overlap between the two modifications was mainly related to gene expression, chromatin organization, chromatin assembly, mRNA binding, and histone binding processes. Moreover, we identified and validated WSTF and LMNA, which were both acetylated and phosphorylated proteins upon SAHA treatment in NPC cells.

Williams syndrome transcription factor (WSTF), which is a transcription factor and tyrosine kinase, had been reported to involve in cancer development. Previous studies showed that both acetylation and phosphorylation at specific sites of WSTF regulated its oncogenic functions, either inhibitory or stimulatory (Wang et al., 2016b; Liu et al., 2020a). Here, we showed for the first time that SAHA modulated phosphorylation at Ser349/158 and acetylation at K1335 of WSTF, which may result in the anti-tumor effects of SAHA in NPC cells. Lamin A (LMNA) protein, belonging to the lamins family, is a nuclear lamina structural protein determining the nuclear shape and size. In addition, it has been revealed that the expression and function of LMNA were aberrant in several cancers, such as colorectal cancer, liver cancer, brain cancer, and breast cancer (Liu and Ikegami, 2020). Meanwhile, lamins were thought to involve in many cellular processes, including transcriptional regulation, DNA damage response, and cell cycle regulation (Liu et al., 2020b). Similar to many other proteins, PTMs, for example, phosphorylation and acetylation, affected LMNA function. Evidence suggested that phosphorylation of LMNA at specific sites, such as Ser22, and Ser392, mainly regulated gene transcription. In the present study, we reported that SAHA increased phosphorylation of LMNA at the S390 site, while declined its acetylation of K270/311 residues in NPC cells. In agreement with our study, Peter C et al found that phosphorylation and acetylation were synergistic coupling in response to EGF stimulation, and H3 phosphorylation can affect the efficiency of acetylation reactions (Cheung et al., 2000). To obtain a deeper insight into the anti-tumor activity of SAHA in NPC cells, how phosphorylation and acetylation of WSTF and LMNA interact will be investigated in the future.

## Conclusion

In this study, taking the advantages of TMT labeling, TiO_2_ enrichment, acetylated antibody enrichment, and high-resolution LC-MS/MS, we presented a large-scale quantitative analysis of the global proteome, acetylome, and phosphoproteome in NPC cells in response to SAHA treatment. Our work provided a precious database that will contribute to the development of predictive and personalized practice in NPC. Furthermore, our study investigated the underlying mechanisms of SAHA’s anti-tumor activity and indicated that SAHA may serve as a novel therapy for NPC patients.

## Data Availability

The datasets presented in this study can be found in online repositories. The names of the repository/repositories and accession number(s) can be found below: ProteomeXchange, accession no: PXD021029.

## Abbreviations

SAHA, suberoylanilide hydroxamic acid; HDACs, histone deacetylases; NPC, nasopharyngeal carcinoma; FDA, Food and Drug Administration; CTCL, cutaneous T-cell lymphomas; EBV, Epstein–Barr virus; PTMs, protein posttranslational modifications; LC-MS/MS, liquid chromatography–tandem mass spectrometry; IMAC, immobilized metal affinity chromatography; TiO_2_, titanium dioxide; DEPs, differentially expressed proteins.
