# Supplementary figures and images for: Suberoylanilide Hydroxamic Acid (SAHA) Treatment Reveals Crosstalk Among Proteome, Phosphoproteome, and Acetylome in Nasopharyngeal Carcinoma Cells
Source: Front Genet. 2022 May 3;13:873840. doi: 10.3389/fgene.2022.873840 (PMC9110868; doi:10.3389/fgene.2022.873840)

HNE3


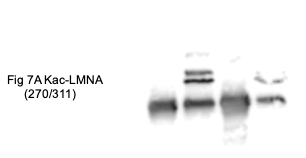

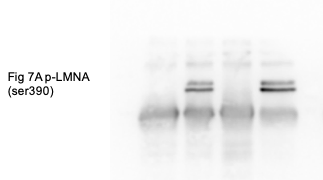


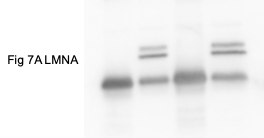

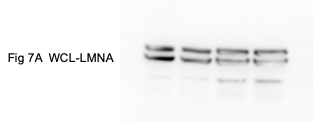


5-8F


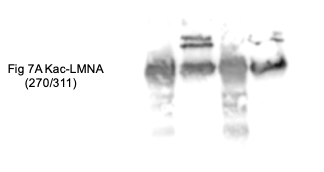

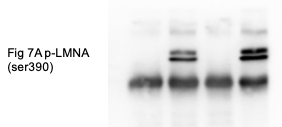

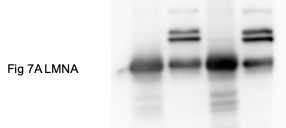

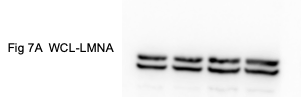


HNE3


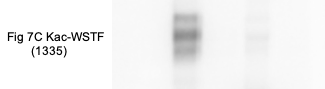

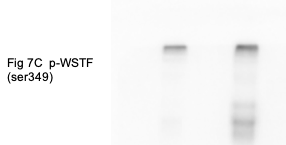


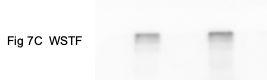

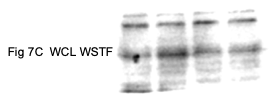


5-8F


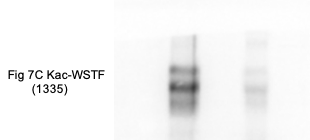

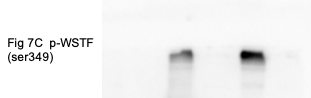


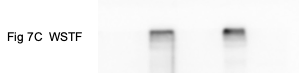

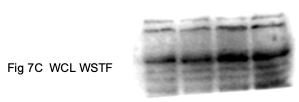

Supplement: Supplementary file 1 [file DataSheet1.DOCX]
